# Exploring every ethical avenue. Commentary: The Moral Obligation to Prioritize Research Into Deep Brain Stimulation Over Brain Lesioning Procedures for Severe Enduring Anorexia Nervosa

**DOI:** 10.3389/fpsyt.2019.00326

**Published:** 2019-05-09

**Authors:** Ludvic Zrinzo, James Wilson, Marwan Hariz, Eileen Joyce, Jane Morris, Ulrike Schmidt

**Affiliations:** ^1^Department of Clinical & Motor Neurosciences, UCL Institute of Neurology, London, United Kingdom; ^2^Department of Philosophy, Faculty of Arts & Humanities, UCL, London, United Kingdom; ^3^Department of Clinical Neuroscience, Umeå University, Umeå, Sweden; ^4^North of Scotland Managed Clinical Network for Eating Disorders, Royal Cornhill Hospital, Aberdeen, United Kingdom; ^5^Section of Eating Disorders, Institute of Psychiatry, Psychology & Neuroscience, Kings College London, London, United Kingdom

**Keywords:** deep brain stimulation, stereotactic ablation, capsulotomy, neurosurgery for psychiatric disease, medical ethics, anorexia nervosa, autonomy

Severe enduring anorexia nervosa (SEAN) has the highest mortality rate of all mental disorders, and existing treatments have limited efficacy (1). Therefore, clinical researchers are morally obliged to follow all possible avenues into the development of an effective therapy. A recent Frontiers Perspective is mistaken when it states that “lesioning procedures in (SEAN) are unethical at this stage of knowledge” (2).

This perspective rightly raises concerns that, in a recent Chinese study on capsulotomy for anorexia, patient selection was at odds with published guidelines from Western institutions (3). However, it conflates this issue with the ethics of research into stereotactic ablation for SEAN in general. Moreover, it places exclusive emphasis on the advantages of deep brain stimulation (DBS). Here we highlight a few of the advantages of stereotactic ablation, when compared with DBS, that were glossed over or entirely ignored (**Table 1**). Ethical research into both stereotactic ablation and DBS should be taken forward. It should be for informed patients to decide which they prefer.

**Table 1:** Potential advantages and disadvantages of stereotactic radiofrequency ablation and deep brain stimulation (DBS) for anorexia.

	Stereotactic radiofrequency ablation	Deep brain stimulation
**Mode of anesthesia**	Local anesthetic may be safer in patients with multisystem abnormalities	General anesthetic is required and may carry additional anesthetic risks
**Risk of infection**	Very low risk	Infection may result in withdrawal of therapy if hardware has to be removed
**Hardware-related problems**	No risk	Risk of skin erosion, lead fracture, pulse-generator malfunction resulting in withdrawal of therapy and requiring further surgery
**Hardware maintenance**	No risk	Ethical issues about lifelong surgical aftercare; commitment to further surgical procedures for pulse-generator replacement
**Body image concerns related to surgery**	Less problematic	Potentially more problematic with scars and implanted hardware on torso
**Adverse neuropsychological effects**	Potential for irreversible changes	Likely to be reversible
**Reversibility**	Not reversible	Early reversibility; potential for long-term DBS “dependence” and symptom rebound
**Issues of “control”**	Some individuals may not want to undergo stereotactic ablation	Implanted device may be seen as a form of “physician control”; others may feel actively involved in making ongoing decisions
**Specialist follow-up**	One-off surgical procedure; follow-up by anorexia team	Considerable commitment to lifelong follow-up in centralized functional neurosurgery center plus follow-up by anorexia team
**Cost**	Relatively cheap	Relatively expensive; less likely to become widely available to patients, even if shown to be effective

The number of patients reported to have undergone DBS for anorexia is small, making it very difficult to comment on the possible spectrum of adverse events in practice. The largest series to date included 16 patients; reported adverse events were seizures, air embolism, hardware infection, and malfunction (4).

Other than a single case report (with no complications), the experience of capsulotomy in patients with anorexia is limited to the aforementioned Chinese study; in a series of 74 patients, disinhibition, memory loss, and lethargy were reported but without any indication of the degree of severity (3, 5). The risk of adverse neuropsychological effects is often cited by psychiatrists as the main reticence for referral for ablative stereotactic procedures. However, a closer look at capsulotomy in other mental disorders may suggest otherwise. Studies that include detailed neuropsychological testing before and after capsulotomy in obsessive compulsive disorder (OCD) and depression often report improvement (fluency, inhibition function, set-shifting, decision-making, and IQ scores) (6, 7) or no significant change (personality testing, executive functions, memory, concentration, and attention) (7, 8) in neuropsychological domains. Indeed, when neuropsychological side effects do occur (memory problems, cognitive impairment, personality change), they appear to be associated with large, dorsally placed lesions and excessively high radiation doses during gamma knife capsulotomy (9–11). Clinical and neuroimaging studies suggest that ventrally placed lesions in the anterior capsule provide good efficacy with low rates of adverse effects (7, 11).

One of the basic ethical principles of research is to minimize risk to participants. While DBS has an excellent safety track record, stereotactic ablation may carry less surgical risk in anorexia patients. Ablation can be performed under local anesthesia without the need for general anesthesia—a significant advantage when multisystem abnormalities carry important anesthetic implications (12). Additionally, DBS carries a risk of hardware infection or skin erosion and has a much greater cosmetic impact than ablation, an important factor for some individuals with SEAN and body image concerns. Moreover, monitoring and follow-up in centralized specialist services involves considerable commitment of travel time, effort, and expense for these potentially frail vulnerable patients, some of whom live in remote and rural areas.

Much emphasis is placed upon the “reversibility” of DBS, which is definitely true of the first few weeks or months. However, we must also acknowledge that DBS is *not* reversible after a few months or years. For example, switching off DBS in patients with obsessive compulsive disorder (OCD) can result in deterioration of affective symptoms that exceed presurgery levels (13). Switching off DBS in patients with Parkinson’s disease or dystonia can lead to fatal parkinsonian or dystonic crisis (14–17). While stereotactic ablation is a one-off procedure, the risk of rebound or recurrence of symptoms makes DBS a lifelong commitment, raising additional ethical issues about lifelong surgical aftercare. Who assumes responsibility for pulse-generator replacement or repair of device malfunction after the trial has closed and the investigators have left the trial institution?

The high cost of DBS as compared to ablation raises other ethical issues. Currently, evidence from randomized controlled trials supports both stereotactic ablation (18) and DBS in the management of OCD (19–21). Nevertheless, stereotactic ablation is the only surgical therapy for OCD currently available within the British National Health Services. Cost is likely to be at least one of the factors leading to this situation. One could argue that it is more ethical to perform research into a therapy that is more likely to come to those who need it, whether within the UK or indeed globally. DBS is beyond the financial reach of most countries and individuals.

The Frontiers Perspective states: “there are no published systematic comparisons between DBS and ablative neurosurgery for any psychiatric indication.” This is not the case. Indeed, a recent review of surgery for OCD suggests that capsulotomy provides a number of advantages over DBS at the same anatomical target (22). In one study, two of three patients whose OCD symptoms were refractory to DBS subsequently responded to stereotactic ablation at the same anatomical target (19). Therefore, a negative trial of DBS does not discount the possibility of a positive trial of stereotactic ablation at the same target, further supporting concurrent research into both surgical approaches.

Reference is made to a case report of capsulotomy in an individual with comorbid anorexia nervosa and OCD who experienced significant improvement in both conditions (5). Bizarrely, this is used as evidence against stereotactic ablation since, at the 3-month time point, the patient felt negatively about the procedure, despite being very positive about the surgery at the 1-year time point. It is not at all uncommon for patients to be ambivalent about having undergone surgery in retrospect, even after well-established (irreversible) surgical procedures. Clearly, this is not a good reason to discontinue the approach as long as comprehensive informed consent is part of the process.

The Perspective piece suggests that DBS is “a dynamic process in which the patient is actively involved in making on-going decisions.” Individuals who value this approach may opt to participate in a trial of DBS. However, others might prefer the option of a single procedure without the need for repetitive hospital appointments to “tweak” stimulation or of committing to further inevitable surgical interventions. Such persons would also be exercising active choice when choosing to enroll in a trial of stereotactic ablation that is governed by ethical safeguards to firmly ensure that they would not submit passively.

It has been highlighted that individuals with SEAN place great value on control. Whether an individual is contemplating enrolment in a trial of DBS or stereotactic ablation, a fully informed adult, with capacity to give consent, can exercise that control by agreeing or declining to participate in that particular study. Freely making long-term or irreversible commitments is itself an important exercise of autonomy. Thus, preventing SEAN patients from making irreversible choices in the name of protecting their control could be a form of disrespect for autonomy.

The Perspective presents the very valid opinion of a self-selected group of individuals who have agreed to participate in a trial of DBS. However, when we conducted focus groups with SEAN individuals, opinions included those against any sort of surgery, some interested in DBS, and others positive about entering a trial of stereotactic ablation (unpublished work). “Many with SEAN have few options remaining to them and feel hopeless.” Trying to restrict surgical research in SEAN to DBS places further limits on the choice of individuals who place great value on control, have few options, and feel hopeless.

Finally, we wish to emphasize that we fully support ethical research into DBS for SEAN and for other mental disorders. Indeed, while our group provides a national stereotactic ablation service for severe refractory OCD and depression, we have also conducted a number of trials of DBS in mental disorders (23–25). We have considerable experience of the potential advantages and disadvantages of both approaches. It is true that stereotactic ablation raises ethical considerations over and above those raised by DBS. However, as demonstrated above, the converse is also true.

Ultimately, well-informed adult participants with capacity should be free to decide whether or not they wish to participate in a particular ethically approved research project. Individuals with SEAN might view the balance of risks of stereotactic ablation as preferable to DBS in their particular situation. It is inappropriately paternalistic to dictate otherwise.

We applaud the Oxford group for their pioneering work on DBS for SEAN in the UK. However, bias toward research into DBS has no rational, moral, or ethical basis. Suppressing either of the surgical approaches at this stage of research is potentially damaging to research in a field that is in desperate need of novel and effective therapies.

## Author Contributions

LZ, JW, MH, EJ, JM, and US were all involved in the initial conceptual design of the paper. LZ developed an initial draft. All authors each collaboratively reviewed and edited the manuscript for intellectual content.

## Conflict of Interest Statement

The authors declare that the research was conducted in the absence of any commercial or financial relationships that could be construed as a potential conflict of interest.
